# Global humid tropics forest structural condition and forest structural integrity maps

**DOI:** 10.1038/s41597-019-0214-3

**Published:** 2019-10-25

**Authors:** Andrew Hansen, Kevin Barnett, Patrick Jantz, Linda Phillips, Scott J. Goetz, Matt Hansen, Oscar Venter, James E. M. Watson, Patrick Burns, Scott Atkinson, Susana Rodríguez-Buritica, Jamison Ervin, Anne Virnig, Christina Supples, Rafael De Camargo

**Affiliations:** 1 Landscape Biodiversity Lab, Ecology Department, Montana State University, Bozeman, MT 59717 USA; 2 Global Earth Observation & Dynamics of Ecosystems Lab, School of Informatics, Computing, and Cyber Systems, Northern Arizona University, Flagstaff, AZ 86011 USA; 3 Global Land Analysis and Discovery, University of Maryland, College Park, MD 20740 USA; 4 Conservation Solutions Lab, University of Northern British Columbia, Prince George, BC Canada; 5 School of Earth and Environmental Sciences, University of Queensland, Queensland, Australia; 6 Wildlife Conservation Society, Global Conservation Program, Bronx, NY 10460 USA; 7 United Nations Development Programme, One United Nations Plaza, New York, NY 10017 USA; 8 Alexander von Humboldt Biological Resources Research Institute, Bogotá, Colombia; 9 LCE - Laboratoire Chrono-Environnement, Université Franche-Comté, UMR 6249 - CNRS-UFC, Besançon, 25000 France

**Keywords:** Macroecology, Forestry, Ecosystem services, Conservation biology, Biodiversity

## Abstract

Remotely sensed maps of global forest extent are widely used for conservation assessment and planning. Yet, there is increasing recognition that these efforts must now include elements of forest quality for biodiversity and ecosystem services. Such data are not yet available globally. Here we introduce two data products, the Forest Structural Condition Index (SCI) and the Forest Structural Integrity Index (FSII), to meet this need for the humid tropics. The SCI integrates canopy height, tree cover, and time since disturbance to distinguish short, open-canopy, or recently deforested stands from tall, closed-canopy, older stands typical of primary forest. The SCI was validated against estimates of foliage height diversity derived from airborne lidar. The FSII overlays a global index of human pressure on SCI to identify structurally complex forests with low human pressure, likely the most valuable for maintaining biodiversity and ecosystem services. These products represent an important step in maturation from conservation focus on forest extent to forest stands that should be considered “best of the last” in international policy settings.

## Abstract (summary)

**Table:** 

Measurement(s)	forested area
Technology Type(s)	digital curation
Sample Characteristic - Environment	forest biome
Sample Characteristic - Location	South America • Africa • Asia

Machine-accessible metadata file describing the reported data: 10.6084/m9.figshare.9885341

## Background & Summary

The value of forests for biodiversity, ecosystem services, and human well-being is well established^1,2^. Consequently, much effort has focused on mapping forests globally and assessing changes in their extent. Remotely sensed forest metrics are continually evolving from forest presence, to measures of forest loss and gain, height, and landscape pattern (Table 1). The global earth observation community has called for the integration of such metrics into Essential Biodiversity Variables (EBVs) that distinguish the ecological quality of forests with regards to ecosystem structure, function, and composition^3^. We introduce two forest indices as candidate EBVs that quantify forest structure and human pressure.

**Table 1:** Forest metrics developed for various conservation applications in previous studies and those presented in this paper (lower two rows).

Forest Characteristic	Input forest metrics	Data source	Extent, resolution	Reference
Forest extent	Forest presence	AVHRR	Africa, 1 km	Tucker *et al*.^40^
		AVHRR	Global, 1 km	Loveland *et al*. ^41^
Forest Intactness	Forest presence	AVHRR	Global, 1 km	Bryant *et al*.^42^
	Human footprint	Various		Wade *et al*.^43^
Forest loss/gain	Forest presence threshold	NOAA 7	Amazonia, 1 km	Woodwell *et al*.^44^
		Landsat	Amazonia, 30 m	Skole and Tucker^45^
	Forest presence threshold Canopy cover (%)	Landsat	Global, 30 m	Hansen *et al*.^25^
Forest stature	Canopy height	GLAS	NW US, 15 km samples	Lefsky^46^
		GLAS Landsat	Sub-Saharan Africa, 30 m	Tyukavina *et al*.^27^ Hansen *et al*.^26^
Large intact landscape	Canopy cover (%), Canopy change threshold	MODIS	Global, 30 m	Potapov *et al*.^47^
Hinterland forest	Human pressure Edge distance Patch size	Landsat	Global, 30 m	Potapov *et al*.^48^ Tyukavina *et al*.^49^
Forest Structural Condition Index	Tree cover (%), Loss Year Canopy height	Landsat GLAS	Humid tropics, 30 m	This paper
Forest Structural Integrity Index	Structural Condition Index Human Footprint	Landsat GLAS Various	Humid tropics, 30 m	This paper

The Forest Structural Condition Index (SCI) quantifies canopy stature, cover and disturbance history across the humid tropics. The SCI is derived from canopy cover, canopy height, and time since forest loss (Table 1). The index spans from short, open-canopy, recently disturbed forests to tall, closed canopy stands that have not been disturbed since 2000. Forest stature and canopy cover are products of both the biophysical potential of a local site and of disturbance history^4,5^. The tallest, most dense forests are found in settings with favorable climate and soils but with low levels of natural or human disturbance. Our maps of SCI are the first to identify locations in the humid tropics of tall, dense forests resulting from high biophysical potential and low disturbance rates. The SCI was validated against estimates of foliage height diversity derived from airborne lidar across gradients in forest structure from recently disturbed forests, plantations, older secondary forest, and primary forest.

We overlay the updated human footprint^6^ on the SCI to derive the Forest Structural Integrity Index (FSII). The name is consistent with the concept of ecological integrity defined as a “system’s capacity to maintain structure and ecosystem functions using processes and elements characteristic for its ecoregion^7^” or “a measure of the composition, structure, and function of an ecosystem in relation to the system’s natural or historical range of variation, as well as perturbations caused by natural or anthropogenic agents of change^8^”. Accordingly, the FSII is based on the structural complexity of a stand relative to the natural potential of the ecoregion and level of human pressure. Forests of high structural integrity are relatively tall, high in canopy cover, older, and with relatively low human pressure.

We developed the SCI and the FSII to aid signatory countries of the Convention on Biodiversity in quantifying success towards meeting the Aichi Biodiversity Targets (ABTs) of the 2011–2020 Strategic Plan for Biodiversity for reducing forest loss and fragmentation and for increasing connectivity among protected areas. We anticipate broader applications of these indices as predictors of or surrogates for biodiversity and ecosystem services given that forest structure is a key determinant of niche diversity and species diversity^9–11^. Thus, the SCI is expected to better predict habitat suitability for forest-structure dependent species and community richness than forest presence or forest intactness. Similarly, carbon storage is known to increase with forest height, stem density, and stand age^12,13^, and forests with high SCI are likley to be especially important for studies of carbon accounting. Hydrological flows from watersheds are influenced by forest structucture in complex ways relating to soil infiltration, groundwater recharge, and subsurface and groundwater flows^14^. The SCI can therefore be used to test hypotheses on relationships between forest structure and water yield.

The FSII has high potential for applications in conservation assessment and planning. An increasing number of studies have shown that human pressure in various forms can have negative effects on native species^15–21^. High integrity forests may be uniquely important for conservation because they support species and processes that require well-developed forests and are sensitive to human activities^22^. By intersecting structural condition with human pressure, the FSII identifies the subset of forests that are likely high in quality for biodiversity and production of ecosystem services. Thus, stands of high FSII represent “the best of the last” and represent an important subset of remaining forests that are especially important for conservation planning.

## Methods

### Study area

**Fig. 1:**
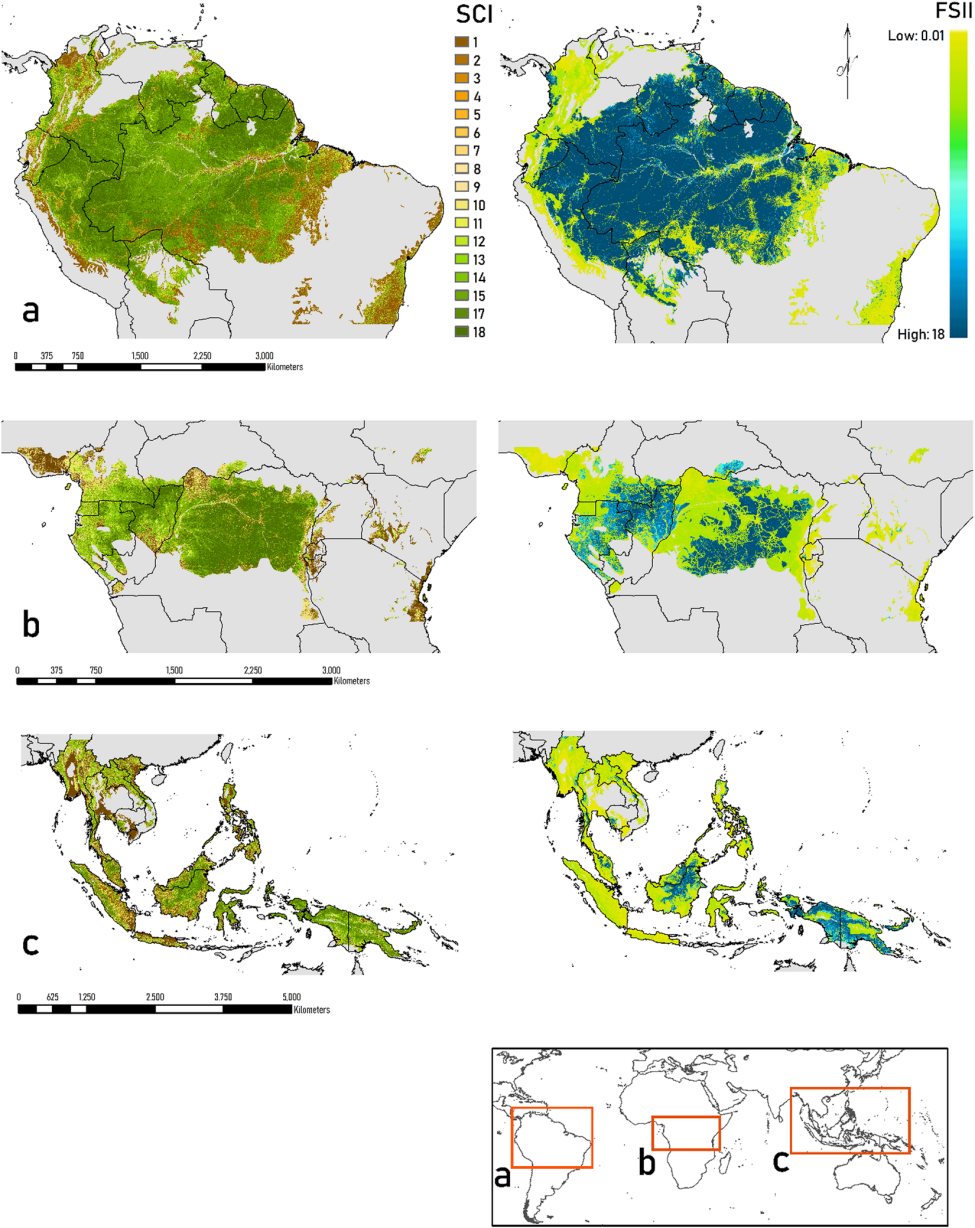
Maps of SCI and FSII across the global moist broadleaf biome. (**a**–**c**) Distribution of forest structural condition and forest structural integrity for the South America, Africa, and southeast Asian project areas.

The project area (Fig. 1) focuses on the humid tropical forests of South America, Africa, and Asia. SCI and FSII were mapped within the Resolve 2017 Tropical & Subtropical Moist Broadleaf Biome^23^. The project area includes the Landsat scenes within 10°N and 20°S in South America, 10°N and 10°S in Africa, and 30°N and 10°S in Southeast Asia. These forests tend to have relatively rapid rates of succession and rapid canopy recovery following disturbance^24^. Thus, the 17-year record of time since disturbance is meaningful for distinguishing recently disturbed from recovering forests in this region.

The project area includes complex gradients in climates, landform, and soils that influence natural and human disturbance regimes, forest growth rates, and forest structure. In South America, the project spans from Pacific coastal rain forests eastward across the Andes montane forests, and across the Amazon lowland humid forests. In Africa, the humid forests lie between the drier biomes of the Sahel to the north and savanna biomes to the south. Southeast Asia has sharp coastal to mountain gradients that support lowland, wetland, and montane moist forests.

### Input data sets

The SCI was derived from three data sets: (1) global tree cover in 2010^25^, (2) forest loss between 2000–2017^25^, and (3) canopy height in 2012^26^ (Table 2). Tree cover and forest loss were derived from growing-season Landsat 7 and 8 Enhanced Thematic Mapper Plus (ETM+) data processed in Google Earth Engine (GEE)^24^. Training data to relate to the Landsat metrics were derived from image interpretation methods, including mapping of crown/no crown categories using very high spatial resolution data such as Quickbird imagery, lidar canopy heights derived from spaceborne Geoscience Laser Altimetry System (GLAS) and airborne lidar data, existing percent tree cover layers derived from Landsat data, and global MODIS percent tree cover, rescaled using the higher spatial resolution percent tree cover data sets. On-screen image interpretation was used to delineate change and no change training data for forest cover loss and gain. Percent tree cover, forest loss and forest gain training data were related to the Landsat time-series metrics using a decision tree classifier. Outputs include percent tree cover, forest loss, and forest gain from 2000 to 2017 at a 30 m spatial resolution. Trees were defined as all vegetation taller than 5 m in height. Forest loss was defined as a stand-replacement disturbance or the complete removal of tree cover canopy at the Landsat pixel scale. More specifically, pixels changing from >50% crown cover to 0% crown cover were defined as loss plots. Selective removals from within forested stands that did not lead to a non-forest state, however, were not included in the change characterization. In this application we use the forest loss metric but not the forest gain product.

**Table 2:** Characteristics of the input data layers used to derive the SCI and FSII Index.

Name	Derived from	Resolution	Validation	Reference
Tree cover	Landsat	30 m 2010	Image interpretation of 1500 samples per biome globally	Hansen *et al*.^25^
Loss year	Landsat	30 m 2000–2017		
Canopy height	GLAS/Landsat	30 m 2012	Product comparison to GLAS training data in Sub-Saharan Africa	Hansen *et al*.^26^
Human footprint	Built environments, Population density, Electric infrastructure, Croplands/pasture, Railways/roadways, Navigable waterways	1 km 2013	3114 × 1 km^2^ random sample plots globally	Venter *et al*.^6^

The validation exercise for the global forest change metrics was performed independently of the mapping exercise^24^. Areas of forest loss and gain were validated using a probability-based stratified random sample of 1,500 blocks of 120 m per biome using image interpretation of time-series Landsat, MODIS and very high spatial imagery from GoogleEarth. Forest loss estimated from the validation reference data set totaled 2.2 M km^2^ (SE of 0.3 M km^2^) compared to the map total of 2.3 M km^2^. Forest gain estimated from the validation sample totaled 0.9 M km^2^ (SE of 0.2 M km^2^) compared to the map total of 0.8 M km^2^. The mapped data validated especially well against the reference data for tropical forests^24^.

Canopy height in 2012 was derived by the Global Land Analysis & Discovery Lab (GLAD) at the University of Maryland^26^. Mapping tree heights with multi-spectral imagery is a relatively new application and is dependent on integrating synoptic coverage optical data with samples of height data. Optical data are considered to be sensitive to land cover properties in the horizontal plane and relatively insensitive to vertical structure. However, exploiting both the temporal and spectral information domains has advanced the use of Landsat in the characterization of vertical vegetation structure. The key to doing so is the availability of suitable calibration and validation data. For example, GLAS data has been used by Tyukavina *et al*. (2015) to train Landsat 7 data to derive tree height^27^. Similarly, Hansen *et al*. (2016) regressed GLAS height data on Landsat time-series multi-spectral data to estimate tree height from a set of multi-temporal metrics from Landsat 7 and 8 for the 2013 and 2014 calendar years in Sub-Saharan Africa^26^. The regression model was validated by using reserved calibration data to determine the ability of the Landsat inputs to recreate the GLAS height calibration data, including height distributions across the study area. The regression tree algorithm accurately reproduced the GLAS-derived height training data with an overall mean absolute error (MAE) for tree height estimation of 2.45 m. Significant underestimations were quantified for tall tree cover (MAE of 4.65 m for >20 m heights) and overestimations for low/no tree cover (MAE 1.61 for <5 m heights).

We integrated a recently updated global terrestrial human footprint map for 2013^6^. Remotely-sensed and survey information were compiled for built environments, population density, electric infrastructure, crop lands, pasture lands, roads, railways, and navigable waterways. To facilitate comparison across these forms of human pressure, each was transformed to a 0–10 scale and weighted within that range according to estimates of their relative levels of human pressure following Sanderson *et al*.^28^. The resulting standardized pressures were then summed together to create the human footprint map. The results were validated by comparison with data from visual interpretation of human pressure from high resolution images in 3460 × 1 km^2^ sample plots randomly located across the Earth’s non-Antarctic land areas^6^. Strong agreement was found between the human footprint measure of pressure and pressures scored by visual interpretation of the high-resolution imagery. A root mean squared error analysis found an average error of approximately 13%. An analysis using the Cohen kappa statistic of agreement found that 88.5% of the sample plots were within 20% agreement of the predicted human footprint score.

### SCI classification

Tree cover, loss year, and canopy height were combined to create the SCI (Table 3). The reference year is 2013, with canopy cover from 2010, forest loss expressed as year of loss prior to 2017, and canopy height for 2012. The index ranges from 1 to 18, with the lowest value assigned to stands less than 5 m tall, disturbed since 2012 or with canopy cover less than 25%. The highest value is for stands not undergoing loss since 2000 that are tall in stature and closed canopy.

**Table 3:** Forest structural condition index (SCI) classification scheme. Forest height is from 2012, canopy cover is from 2010, and loss year is for 2001 to 2017. Table values are SCI weights which range from 1 (low SCI) to 18 (high SCI).

Loss Year	Forest height (m)										
	Canopy cover (%)	0–5	>5–15			>15–20			>20		
			Canopy cover (%)			Canopy cover (%)			Canopy cover (%)		
	<25		25–75	>75–95	>95	25–75	>75–95	>95	25–75	>75–95	>95
2013–2017	1	1	1	1	1	1	1	1	1	1	1
2001–2012	1	1	2	3	4	5	6	7	8	9	10
<=2000	1	1	10	11	12	13	14	15	16	17	18

The SCI is designed to be consistent with successional stages of forest development (Table 4). Following canopy replacement disturbance, new stems establish and undergo rapid growth due to abundant resources. Some stems are outcompeted and die as canopy closure is reached and resources become limiting. As some canopy trees reach maturation and die, understory redevelopment occurs in canopy gaps. Gap dynamics become the primary process in the final Old-Growth stage. In tropical rain forests, these stages are often referred to as pioneer, young secondary, old secondary, and primary^29^. In most moist tropical forests, the stem exclusion stage is reached in 5–10 years and the understory re-initiation stage in 20–40 years^23^. Thus, the <6 years since forest loss class in our structural condition index is likely the stand re-initiation stage. The 6–16 years since forest loss class is likely in the stem exclusion stage. The >16 years since forest loss class has an unknown disturbance history prior to 2000 and these stands may be in the late stem exclusion, understory re-initiation, or old growth stages. The canopy height and canopy cover classes give additional insight into the seral stages with older forests generally being of higher stature and with high canopy closure. Intense forest management may alter the rate and trajectories of successional development, thus gradients in SCI are likely to relate to different forest processes in tree plantations compared to natural forests.

**Table 4:** Processes and structures associated with stages of forest succession. Based on Oliver and Larson^50^ and Franklin *et al*.^51^.

Successional Stage	Key Processes	Key Structures
Stand Initiation	Stand initiating disturbance(s) Establishment of new cohort Colonization by new seed Germination from seed bank Minimal or no nutrient limitations Rapid growth	Live trees 100% live crown rations Debris or slash Legacy trees (live or dead)
Stem Exclusion	Canopy closure Density dependent mortality Competitive exclusion of understory Crown differentiation Lower canopy tree loss Self pruning Nutrient limitations develop	Less than 100% life crown ratios Vertically differentiated canopy Heavily shaded understory
Understory Reinitiation	Density independent mortality Canopy gap initiation Understory redevelopment Establishment of shade tolerant species Maturation of pioneer cohort Canopy elaboration Nutrient limitations persist but lessen	Understory herbaceous layer Shade tolerant cohort Few smaller canopy gaps Standing dead trees Some large woody debris Some uprooted or snapped trees
Old Growth	Canopy gap expansion Uprooting and snapping of large trees Live tree decadence (poor form and disease) Development of large branches Pioneer cohort loss Nutrient limitations decline as organic matter accumulates	Large diameter live trees Large branches Rich epiphyte community Continuous vertical foliar profile More standing dead trees More large woody debris More uprooted or snapped trees Horizontally patchy forest Large gaps Densely regenerating old gaps

### Forest structural integrity index classification

The FSII is derived from overlaying the Human Footprint Index of human pressure on the SCI. Human activities can influence forests in several ways in addition to altering forest structure. Hunting and poaching alters wildlife populations without direct effects on habitat^30,31^. Human settlements, roads, and deforested areas create edge effects that can extend hundreds of meters into adjacent forests^32^. These edge effects include invasive species, livestock and pet effects, altered ecological processes, noise and light^19^. The effects of anthropogenic disturbance on biodiversity may exceed that of deforestation^15^. Integrating human footprint with forest structural condition reveals forests that may be of the highest value for biodiversity and various ecosystem services.

For the FSII (Table 5), human pressure classes and weights were: Low (1), HFP < 4; Medium (5), HFP >= 4 and <=15; and High (10), HFP > 15. These threshold values of HFP are consistent with those identified as being highly relevant to responses of vertebrate species endangerment trends to human pressure^4^. FSII was calculated as:

$$FSII=SCI\ast \frac{1}{Human\,Pressure\,Weight}$$

**Table 5:** Weights of the Forest Structural Integrity Index derived from SCI and the Human Footprint.

SCI Value	HFP Class		
	Low (1)	Med (5)	High (10)
1	0.2	0.2	0.1
2	2	0.4	0.2
3	3	0.6	0.3
4	4	0.8	0.4
5	5	1	0.5
6	6	1.2	0.6
7	7	1.4	0.7
8	8	1.6	0.8
9	9	1.8	0.9
10	10	2	1
11	11	2.2	1.1
12	12	2.4	1.2
13	13	2.6	1.3
14	14	2.8	1.4
15	15	3	1.5
16	16	3.2	1.6
17	17	3.4	1.7
18	18	3.6	1.8

**Fig. 2:**
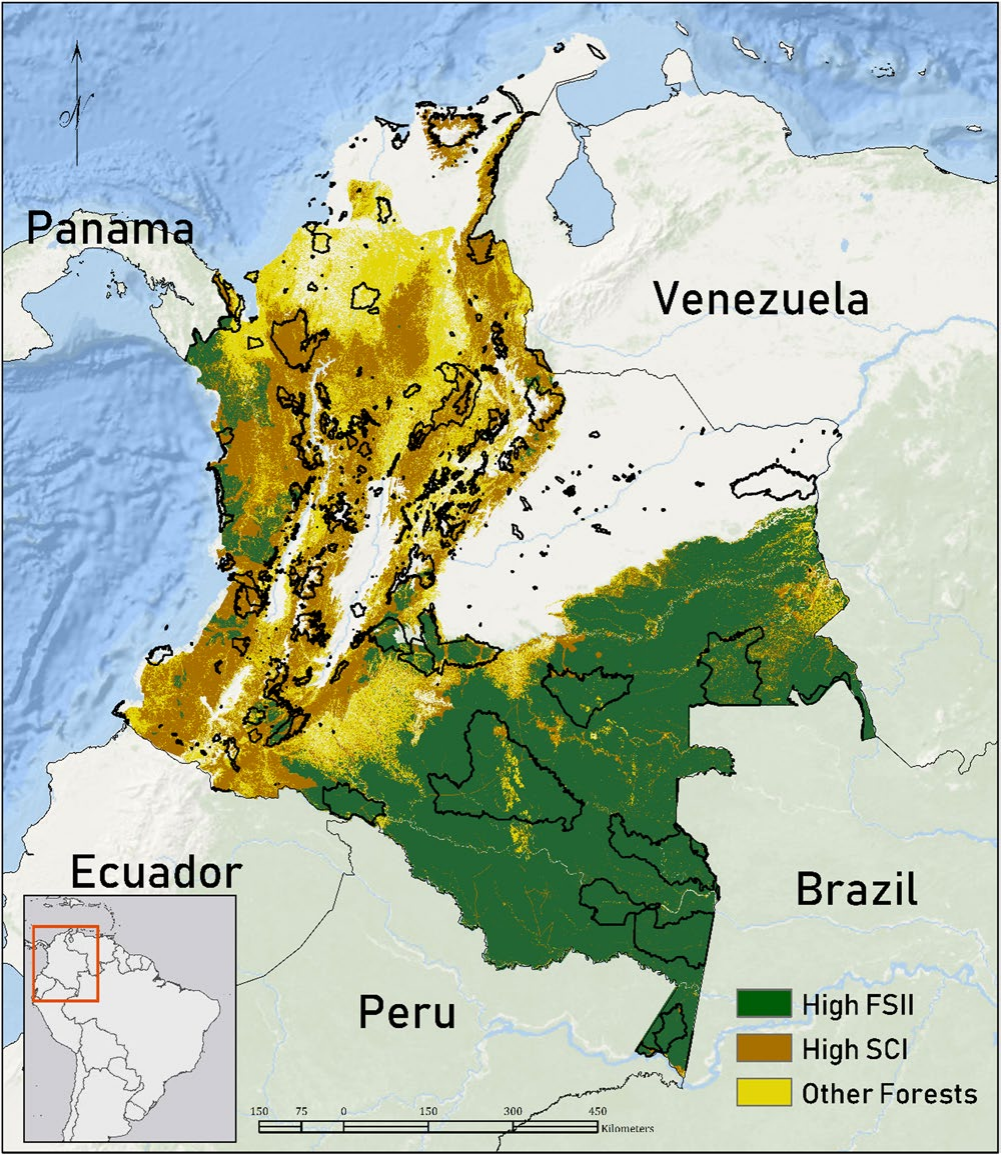
Extent of forests (>25% tree cover), high SCI forests (>=14), and high FSII forests (>=14) relative to protected areas across Colombia.

Resulting values of the FSII range from 0.1 to 18 with the higher values representing forests high in structural complexity and low in human pressure. Forests high in SCI and in FSII represent a small subset forest extent in many ecoregions and sometimes are not present in protected areas (Fig. 2).

## Data Records

The SCI and FSII data sets are distributed as GeoTIFF files through figshare^33^. Each individual input layer (i.e., canopy cover, loss year, canopy height, and human footprint) are also provided in the data record should users wish to alter the weights assigned to derive SCI and FSII in accordance with their particular needs or region. All data are clipped to the moist broadleaf biome of the humid tropics. In addition, the United Nations Development Programme will also make these data available through the UN Biodiversity Lab (www.unbiodiversitylab.org) to enable governments from nearly 140 countries to visualize them in a web-based platform.

## Technical Validation

We performed a quantitative assessment of the extent to which the SCI represents a gradient in forest structure by comparing the SCI values with foliage height diversity (FHD) estimates derived from airborne lidar measurements. Lidar data were acquired by the Sustainable Landscapes Brazil project supported by the Brazilian Agricultural Research Corporation, the US Forest Service, USAID, and the US Department of State.

**Fig. 3:**
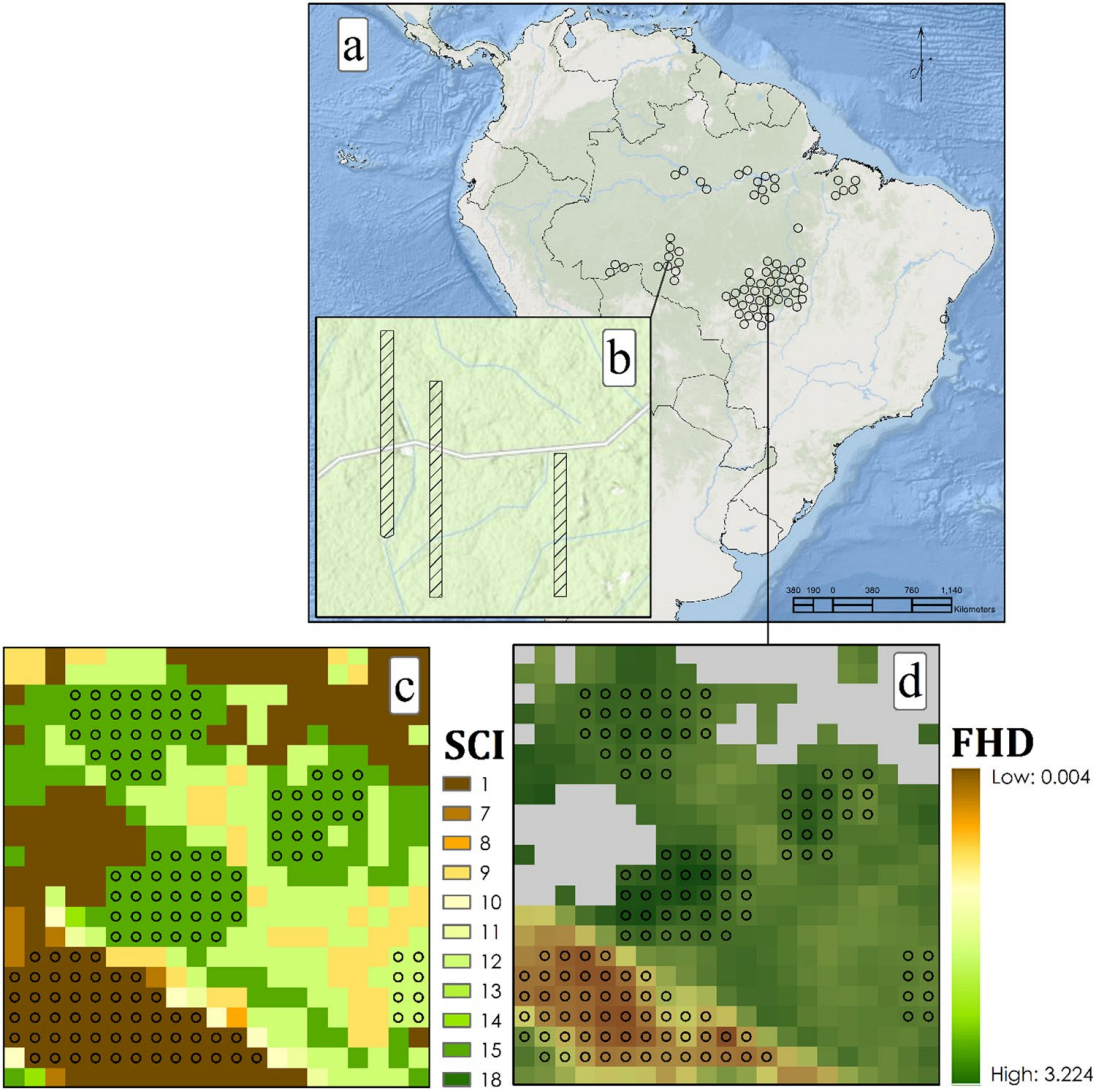
Spatial distribution of the validation procedure. (**a**) Location of lidar transects in Brazil. (**b**) Selected subset of lidar transect footprints. (**c**,**d**) Validation points within homogeneous patches of the SCI and FHD.

The geographic area of the validation was limited to Brazil Sustainable Landscapes lidar transects (Fig. 3) because they are consistent, high quality, and publicly available. As similar lidar datasets become available elsewhere, the geographic scope of the validation will be extended. Patterns of forest structural development in the humid tropics are closely related to disturbance history and seral stage^28^. Thus, the gradient of forest structure in Brazil is representative of that across the humid tropics. Evidence in support of this comes from the patterns of the SCI input data sets across the study area. The range of tree cover, canopy height, and time since disturbance were similar in South America, Africa, and Asia.

We downloaded 72 transect boundaries from the Sustainable Landscapes website (http://www.paisagenslidar.cnptia.embrapa.br/webgis/) and calculated the number of pixels of each SCI class in each transect. For each SCI class, we selected between 5 and 10 transects, with replacement, in which that class was represented. Some classes were rare in the dataset and were only represented by one or two transects.

Only transects that were sampled by airborne lidar since 2010 were included in the validation to best match the sampling period for the SCI. The 72 transects were generally 2–7 km wide by 2–7 km long, although one was as long as 50 km (300 m wide). Lidar points came from the vendor pre-labeled as either ground or vegetation. We used ground points to normalize each vegetation point to height above ground using a k-nearest neighbor, inverse distance weighting algorithm with k = 6 and inverse distance squared as the weighting. Vegetation heights >70 m were filtered out as these are likely caused by clouds or sensor noise. We then calculated point density (points/m^2^) for each 30 m cell. Cells where the lidar point density was relatively sparse (<10 points/m^2^) were omitted from the analysis. For the remaining cells, we calculated FHD using the Shannon index at 1-meter vertical intervals:

$$FHD=-\sum {p}_{i}\,log\,({p}_{i})$$

where, for each 30 m cell, *p*_*i*_ = proportional of vegetation points in the *i*-th vertical interval.

The FHD data set was then reprojected using bilinear interpolation to align with the SCI grids. The SCI layer was filtered to omit cells with a loss year greater than the year of lidar acquisition. Cell alignment between FHD grids in their native projections and SCI grids varied by transect. We corrected for alignment issues by selecting cells within unique homogenous patches of SCI using a minimum patch size of 3 × 3 cells and removing cells within 90 m of the patch perimeter. The result is that validations samples are core areas of homogeneous SCI patches. All point cloud processing was performed with the lidR package in R, v.3.5.2.

We estimated a baseline Ordinary Least Squares (OLS) model and subsequent linear mixed effects models to determine the strength of relationship between SCI and FHD. All models were estimated using the full sample of 114,253 observations. For the mixed effects models, both lidar transect and SCI patch were included as random effects. Lidar transect and SCI patch were identified as random effects due to the hierarchical structure and associated spatial dependencies of the validation data – observations were nested within patches which were grouped into transects. We estimated separate linear mixed effects models to evaluate how imposing alternative random effect structures affected predictive capability. One model included only a random intercept for transect, while the second included a nested random intercept of SCI patch within transect. We report the Akaike Information Criterion (AIC) and R^2^ values^a^^1^ as measures of goodness-of-fit across model specifications.

**Fig. 4:**
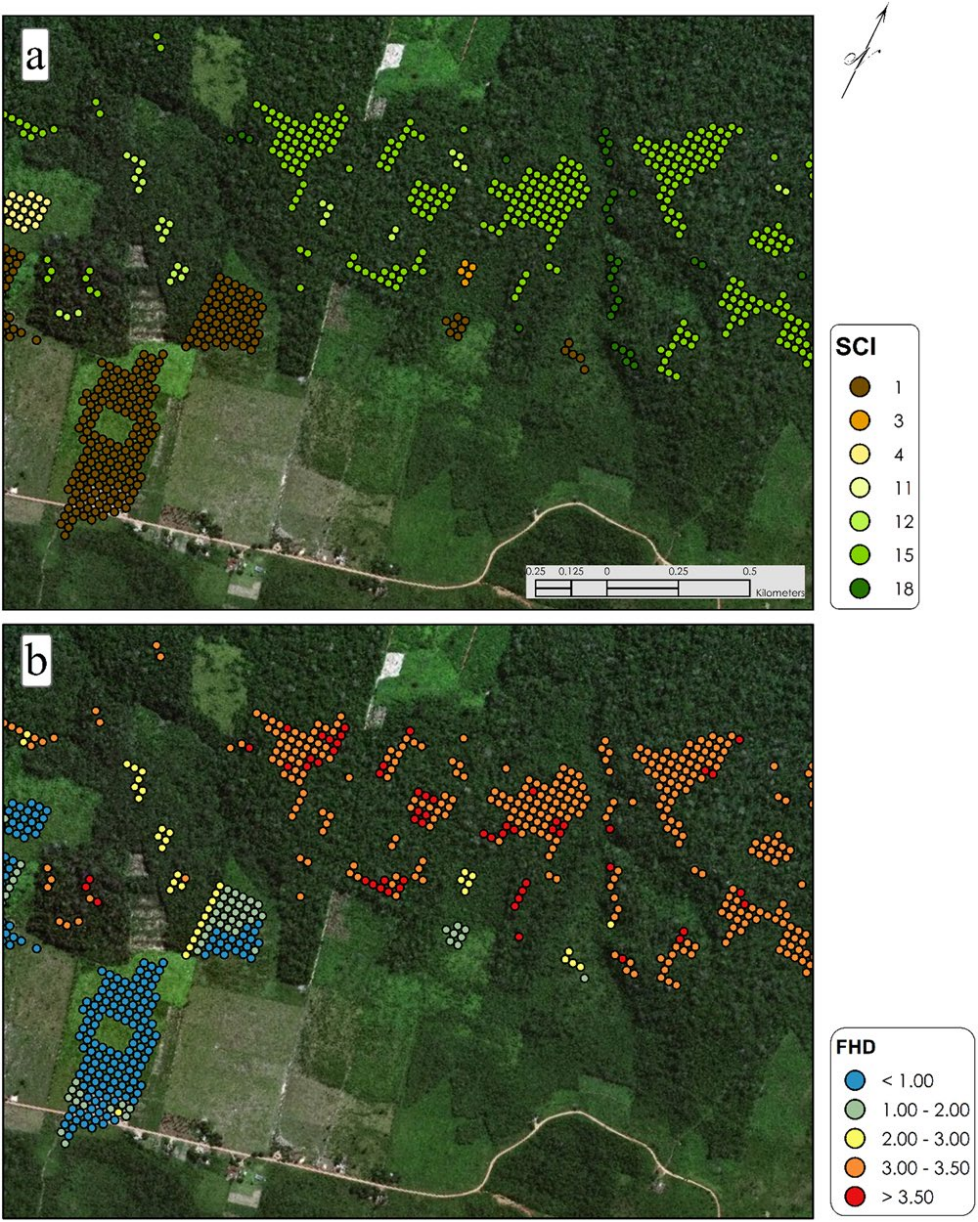
Aerial imagery across a heterogeneous landscape in Brazil. (**a**,**b**) Distribution of SCI and FHD.

**Fig. 5:**
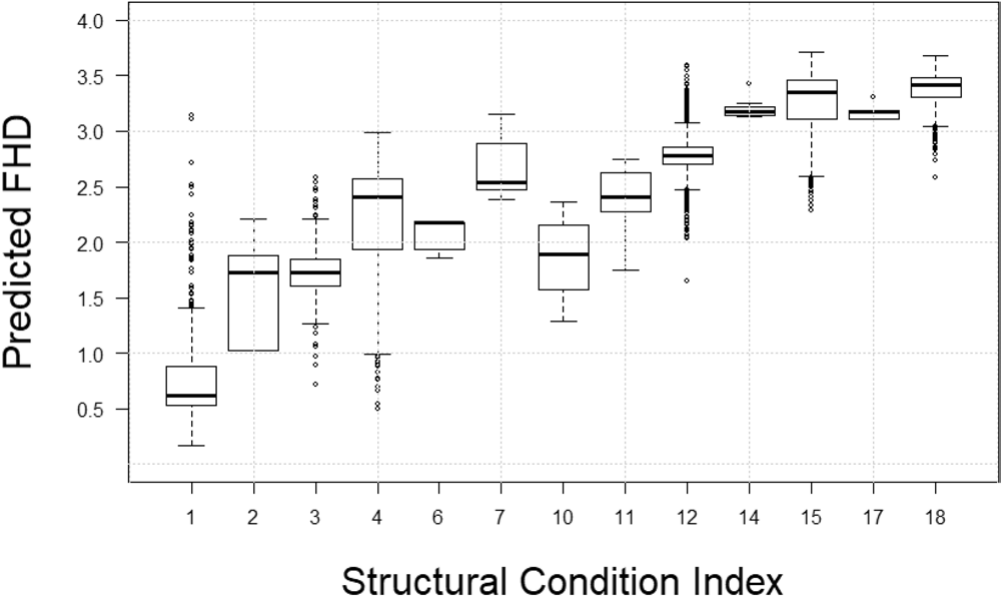
Distribution of predicted FHD for each SCI class.

The ability of the estimated models to predict forest structure was strong across all specifications (Table 6 and Fig. 4). Inclusion of a transect-level random effect significantly decreased AIC relative to the baseline OLS model. Model performance was maximized by including a nested random effect of SCI patch within transect, with SCI explaining 93% of the variation in airborne lidar-derived FHD. Predicted FHD increased with increasing SCI, although variability in predictive capability is evident for particular SCI classes (Fig. 5). This relationship asymptotes at class 14, indicating that SCI is not sensitive to increases in FHD above that level. These findings indicate that SCI is a strong indicator of foliage height diversity, which is known to be an indicator of forest structural complexity^8^.

**Table 6:** Results of model selection for the effects of SCI on FHD.

Model name	Model formula	AIC	R^2^
Baseline OLS	FHD = SCI + ε	47650.92	0.86
Random effect – transect	FHD = SCI + (1|transect) + ε	−11772.14	0.91
Random effect – patch nested in transect	FHD = SCI + (1|transect/patch) + ε	−59424.04	0.93

We additionally evaluated the extent to which the SCI differed among forests of known or inferred stand history. Classes of forest include tree plantations, older secondary forest, and primary forest. Tree plantations have been mapped in Brazil^34^. Defined as “the biophysical presence of trees that are clearly planted and managed by humans”, plantations were located largely based on forest loss and gain data^24^ and confirmed with 2013–14 Landsat imagery and high-resolution imagery. Primary forests were mapped^35^ based on texture analyses of 2001 Landsat data. Primary forest was defined as “mature natural humid tropical forest cover that has not been completely cleared and regrown in recent history”. These forests may include locations that had been selectively logged or had other disturbances that may have altered forest composition and structure but have regrown the canopy texture typical of primary forests. We defined older secondary forest as areas with tree cover >25%, tree height >5 m, not undergoing forest loss after 2000, and not labeled primary forest^35^. Such forests may not have the canopy texture typical of primary forest either because of biophysical factors limiting structural development or because they underwent natural or human disturbance prior to 2000 and have not yet regrown complex structure. We generally expect forest structure to differ among these forest types with canopy cover and height increasing from tree plantations, older secondary forest and primary forest.

Among the validation plots described above, 95% were labeled primary forests, and 2.8% as older secondary forests; none were in tree plantations. We plotted FHD against SCI for these primary forests and older secondary forests. In order to evaluate SCI in tree plantations relative to the other two classes, we plotted frequency distributions of SCI for tree plantations, older secondary forest, and primary forest across the full ecoregions that the validation plots fell within.

**Fig. 6:**
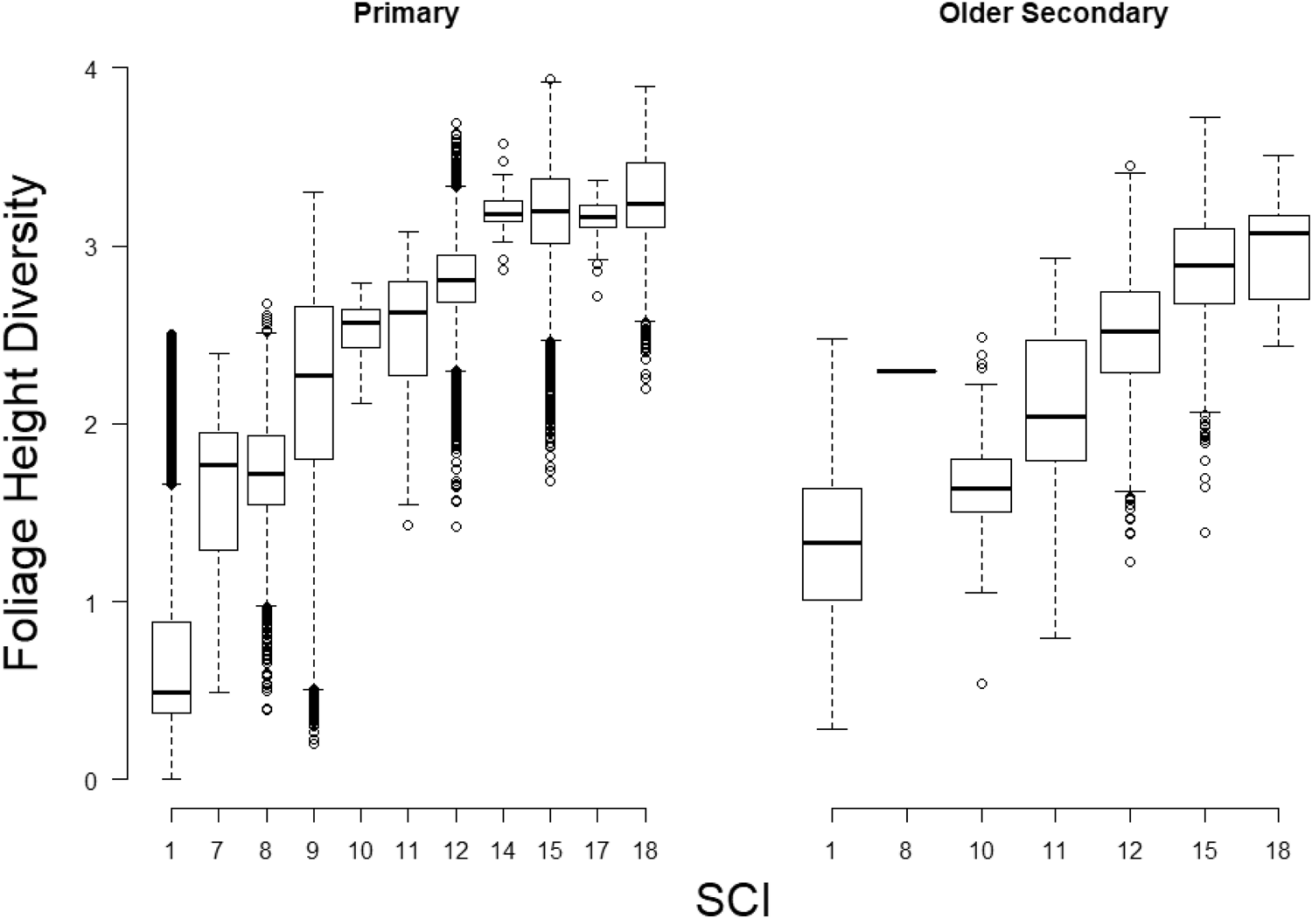
Relationship between SCI and FHD for validation plots labeled as primary and older secondary forest.

Within the validation plots, older secondary forests included a similar range of FHD to primary forests, although maximum FHD in primary forests was slightly higher than in older secondary forests (Fig. 6). These results indicate that some secondary forests can regain over time similar structural complexity to primary forests and may confer many of the ecological functions and ecological services of primary forests^36^. SCI varied with FHD similarly for older secondary forests and primary forests, indicating that SCI is able to distinguish gradients in structural condition equally well in these two forest classes. It is important to note that the highest SCI class (18) includes both primary forest and secondary forest with high FHD. Such secondary forests are likely to have high ecological values similar to primary forest and are important to identify for conservation planning.

**Fig. 7:**
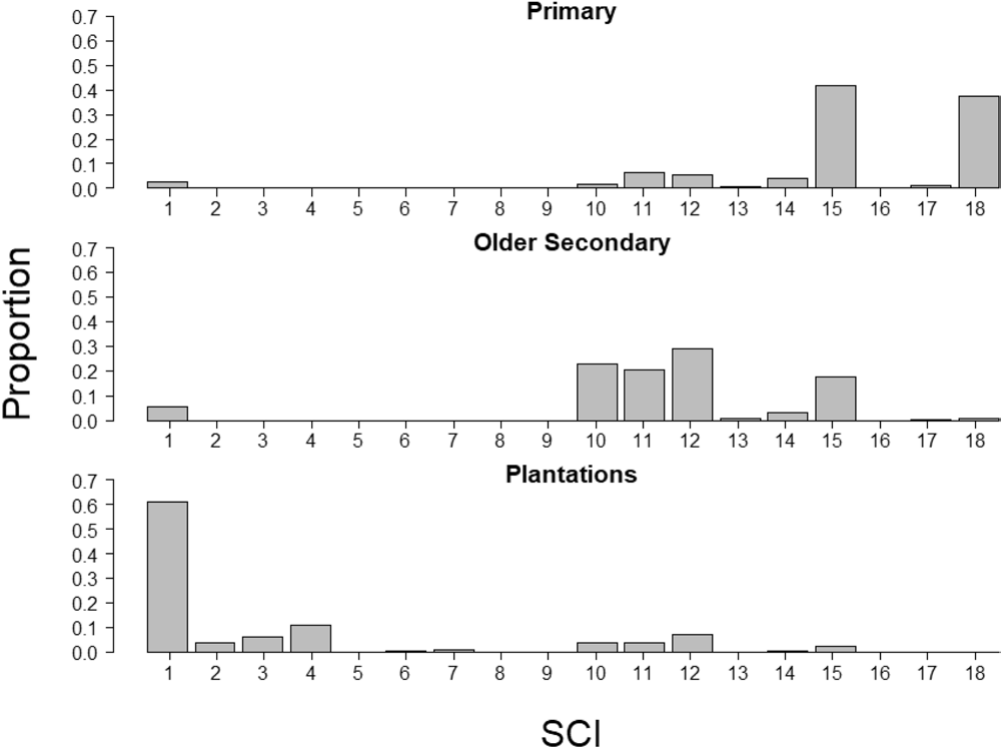
Histograms of SCI for primary forest, older secondary forest, and tree plantations for the ecoregions containing the validation points.

The histograms of SCI show a skew towards higher SCI from tree plantations to older secondary forest to primary forest (Fig. 7). This indicates that the SCI is sensitive to differences in structural condition from the shorter and lower canopy cover tree plantations to the intermediate older secondary forest, to the taller, higher canopy cover primary forests.

## Usage Notes

Mapping structural condition and human pressure on forests represents measures of forest quality that are relevant for studies of species and communities that are habitat specialists^1^. As such, the SCI and FSII maps can be used to inform assessment of remaining forest pattern and conservation planning. These data may be particularly useful for countries that are assessing forest condition with regards to the ABTs under the CBD’s 2020 Strategic Plan for Biodiversity. They are also potentially useful for studies of carbon budgets and hydrology because it is thought that forests with high integrity hold an exceptional confluence of globally significant carbon and hydrological values relative to forests that have experienced damaging human actions. More generally, our approach for quantifying forest integrity based on stand structure may motivate integration of global spatial data layers to represent forest functional integrity and forest compositional integrity^37–39^.

Readers should note that the University of Maryland canopy height data used to develop the SCI and FSII has only been validated in the African tropics as reported in the primary publication^26^. Validation of canopy height for multiple years in 2000–2016 is underway for the three continental tropical areas and is expected to be available in the coming year.

Another important caveat about SCI is that it has an unknown but likely higher level of error at forest/nonforest edges than in patch interiors. This is likely both because of edge effects reducing the classification accuracy of the input data layers and because of error in georegistration of the lidar data set and the SCI data set in the validation. We restricted the validation to non-edge plots to avoid these errors.

A current limitation of the SCI and FSII data sets is that they are static in time (centered on 2013) and cannot currently be used to assess change over time. Efforts are in progress, however, to update the input data layers to allow change analysis for the 2000–2017 period. A second limitation is that FSII has a coarser 1 km resolution compared with the 30 m SCI. This is because of the resolution of the global human footprint data set used to generate FSII. Some countries, such as Colombia, are now using the human footprint methodology to generate national 30 m maps of human pressure and include them in their reports to the CBD on national progress towards the ABTs. When completed, these data could be used to generate FSII at this finer spatial resolution for use in national conservation planning and policy development. A third limitation is that the accuracy of the height data used to generate SCI necessitates height classes being resolved in 5 m height intervals. Canopy metrics resulting from the space-station based GEDI Lidar Mission (which will be available in the coming year) are expected to be much more accurate, ultimately allowing after two more years of data collection a more accurate and useful SCI.

## Data Availability

The SCI and FSII were generated in GEE. The Hansen Global Forest Change v1.5 (2000–2017) data set used to obtain loss year is served by GEE. Tree cover for 2010 was uploaded as a GEE asset from GLAD at the University of Maryland. This was done for each continental portion of the study area using the Python API Earth Engine shell on a parallel processor computer at Northern Arizona University. The same procedure was used for the canopy height data set. Human footprint data were uploaded using the GEE Asset Upload procedure. The SCI weights were generated in GEE by assigning a mask of 0 where conditions are not met and 1 when they are met. An example conditional statement is: var sciImage1 = lossYear.gte(13).and(lossYear.lte(17)).or(forest.lt(25)).or(height.lte(5)). This formulation was then used to multiply the cells with mask = 1 times the appropriate SCI weight. This was done for each combination of input data values for each SCI weight. The resulting maps for each category of SCI weight were combined into a single map using the GEE add command. Similar coding was used for the FSII classification. The resulting SCI and FSII maps for each continent were exported from GEE to Google Drive. GEE code for the classifications are available at Africa: https://code.earthengine.google.com/f46e40ba74164a595f9be5067a7b26cf SE Asia: https://code.earthengine.google.com/e6595f8040af90a7b30a9b89e42c12f6 South America: https://code.earthengine.google.com/285ecd4784d7d4e0bc9a6e6aa966b54c

## Footnotes

1 ^a^A pseudo-R^2^ for the linear mixed effects models using the r2GLMM package in R v.3.5.2.
